# Estimated impact of guidelines-based initiation of dual antihypertensive therapy on long-term cardiovascular outcomes in 1.1 million individuals

**DOI:** 10.1093/ehjcvp/pvae048

**Published:** 2024-07-09

**Authors:** Antonio Coca, Claudio Borghi, George S Stergiou, Irfan Khan, Alexandra Koumas, Jacques Blacher, Mohamed Abdel-Moneim

**Affiliations:** Department of Medicine, University of Barcelona, Hospital Clínic, 143 Casanova st., Barcelona 08036, Spain; University Abat Oliba CEU, 30 Bellesguard St., Barcelona 30905, Spain; Department of Medicine, Science and Surgery, University of Bologna, Bologna, Italy; Hypertension Center STRIDE-7, School of Medicine, Third Department of Medicine, Sotiria Hospital, National and Kapodistrian University of Athens, Athens, Greece; Sanofi, NJ, USA; Axtria Inc., Berkeley Heights, NJ, USA; Hôpital Hôtel-Dieu, AP-HP, Diagnosis and Therapeutic Center, Université Paris Cité; Sanofi, Dubai, United Arab Emirates; Department of Family Medicine, College of Medicine, University of Sharjah, United Arab Emirates

**Keywords:** Hypertension, Clinical practice guidelines, Dual therapy, Simulation, Cardiovascular risk, Event rates

## Abstract

**Aims:**

Guidelines recommend initiation of dual combination antihypertensive therapy, preferably single-pill combination (SPC), in most patients with hypertension. Evidence on narrowing gaps in clinical practice relative to guidelines is limited.

**Methods and results:**

Monte Carlo simulation was applied to 1.1 million patients qualifying for dual combination therapy from a previously conducted retrospective analysis of clinical practice, hospital statistics, and national statistics in the UK. We provide 10-year Kaplan–Meier event rates for the primary endpoint representing a composite of non-fatal myocardial infarction, non-fatal stroke (ischaemic or haemorrhagic), non-fatal heart failure hospitalization, or cardiovascular death. Cox model results from a previously conducted study were utilized to estimate baseline risk, together with evidence on risk reduction from the Blood Pressure Lowering Treatment Trialists’ Collaboration (BPLTTC) meta-analysis and published evidence on blood pressure-lowering efficacy of antihypertensive therapies. In the overall population, estimated 10-year event rates for the primary endpoint in patients with 100% persistence in monotherapy were 17.0% for irbesartan and 17.6% for ramipril. These rates were only modestly better than those observed in clinical practice (17.8%). In patients with 100% persistence in dual therapy, estimated event rates were 13.6% for combinations of irbesartan + amlodipine [absolute risk reduction (ARR) = 8.7% compared with untreated] and 14.3% for ramipril + amlodipine (ARR = 8.0% compared with untreated). The absolute risk of the primary endpoint was reduced by 15.9% in patients with atherosclerotic cardiovascular disease (ASCVD) and 6.6% in those without ASCVD. Similarly, the absolute risk was reduced by 11.7% in patients with diabetes and 7.8% in those without diabetes.

**Conclusions:**

This study represents the first to investigate guidelines-based treatment in hypertensive patients and demonstrates the opportunity for considerable risk reduction by ensuring recommended dual therapy in clinical practice, particularly in the form of SPC with high persistence, relative to no treatment or monotherapy.

## Graphical Abstract

**Graphical Abstract:**
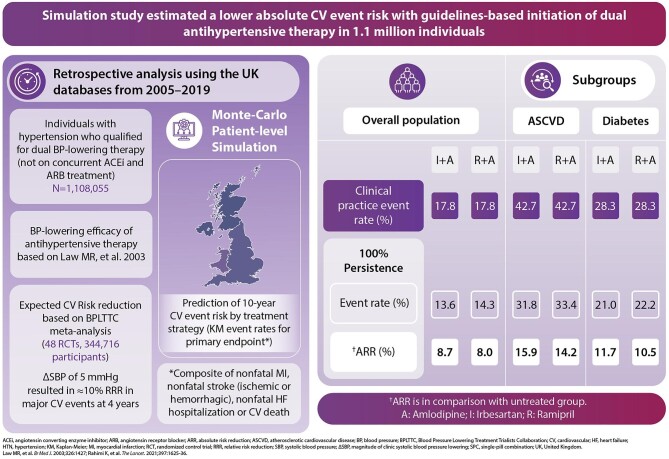
This simulation study demonstrates the opportunity for considerable risk reduction in hypertensive patients by ensuring guidelines-recommended dual blood pressure-lowering therapy in clinical practice, particularly in the form of single-pill combination with high persistence, in individuals with atherosclerotic cardiovascular disease or diabetes, relative to no treatment or monotherapy.

## Introduction

Elevated blood pressure (BP) is a risk factor for incident cardiovascular (CV) disease and recurrent CV events.^1–4^ A large body of evidence from randomized controlled trials (RCTs) demonstrates that initiation of BP-lowering therapy lowers CV risk in a range of at-risk populations, with the risk reduction apparent to achieved BP levels as low as 115 mmHg.^5^ Strategies commonly used in clinical practice for initiating antihypertensive therapy include (i) monotherapy and sequential (stepwise) titration of additional agents as suggested in the 2019 UK National Institute for Health and Care Excellence (NICE) guideline^6^; (ii) dual therapy with multiple agents as free-dose combination (FDC); and (iii) dual therapy with single-pill combination (SPC). Guidelines recommend upfront initiation of dual therapy with two agents [such as angiotensin-converting enzyme inhibitor (ACEi) or angiotensin receptor blocker (ARB) along with a calcium channel blocker (CCB) or diuretic] in most patients. The guidelines also recommend SPC to be preferred as compared with FDC due to implications on improved persistence and adherence.^1–4^

There is insufficient evidence from both clinical trials and observational studies on how a narrowing of gaps in clinical practice relative to contemporary guideline recommendations would translate to clinical benefit at a population level. Only two clinical trials [Avoiding Cardiovascular Events through Combination Therapy in Patients Living with Systolic Hypertension (ACCOMPLISH)^7^ and Combination Therapy of Hypertension to Prevent Cardiovascular Events (COPE)^8^] have directly compared the addition of two drugs in patients with hypertension qualifying for dual combination therapy. It is also useful for stakeholders to identify subgroups who derive the highest clinical benefit from relevant dual therapies representing combinations such as ACE/ARB with CCB (e.g. ramipril with amlodipine or irbesartan with amlodipine). To develop insights into these questions, we conducted a simulation-based analysis to evaluate the 10-year clinical benefit, as measured by the absolute reduction in CV events, of optimal treatment with guidelines-recommended dual agents in comparison to no treatment or monotherapy. The key data supporting the simulation were a population of 1.1 million individuals with hypertension initiating antihypertensive therapy from a previously conducted retrospective analysis of clinical practice, hospital statistics, and national statistics in the UK,^9^ together with evidence from the Blood Pressure Lowering Treatment Trialists’ Collaboration (BPLTTC) meta-analysis,^5^ and published evidence on BP-lowering efficacy of antihypertensive agents.^10^

## Methods

### Study population

The overall population of individuals qualifying for dual BP-lowering therapy as per the 2018 European Society of Cardiology/European Society of Hypertension (ESC/ESH) guideline was identified in the Clinical Practice Research Datalink (CPRD), Hospital Episode Statistics (HES), and Office for National Statistics (ONS) databases in the UK during 2005–2019. Briefly, this population represented those with evidence of clinic systolic BP (SBP) ≥140 mmHg while receiving BP-lowering monotherapy or ≥150 mmHg while untreated (with these points defining index date, or time when the patient first became eligible for dual BP-lowering therapy; index date also represented time zero for follow-up for CV events) and with at least 1-year period of continuous enrolment in the CPRD database prior to index. These databases and the information recorded in them have been used in the derivation and validation of CV risk prediction models.^11,12^

**Figure 1:**
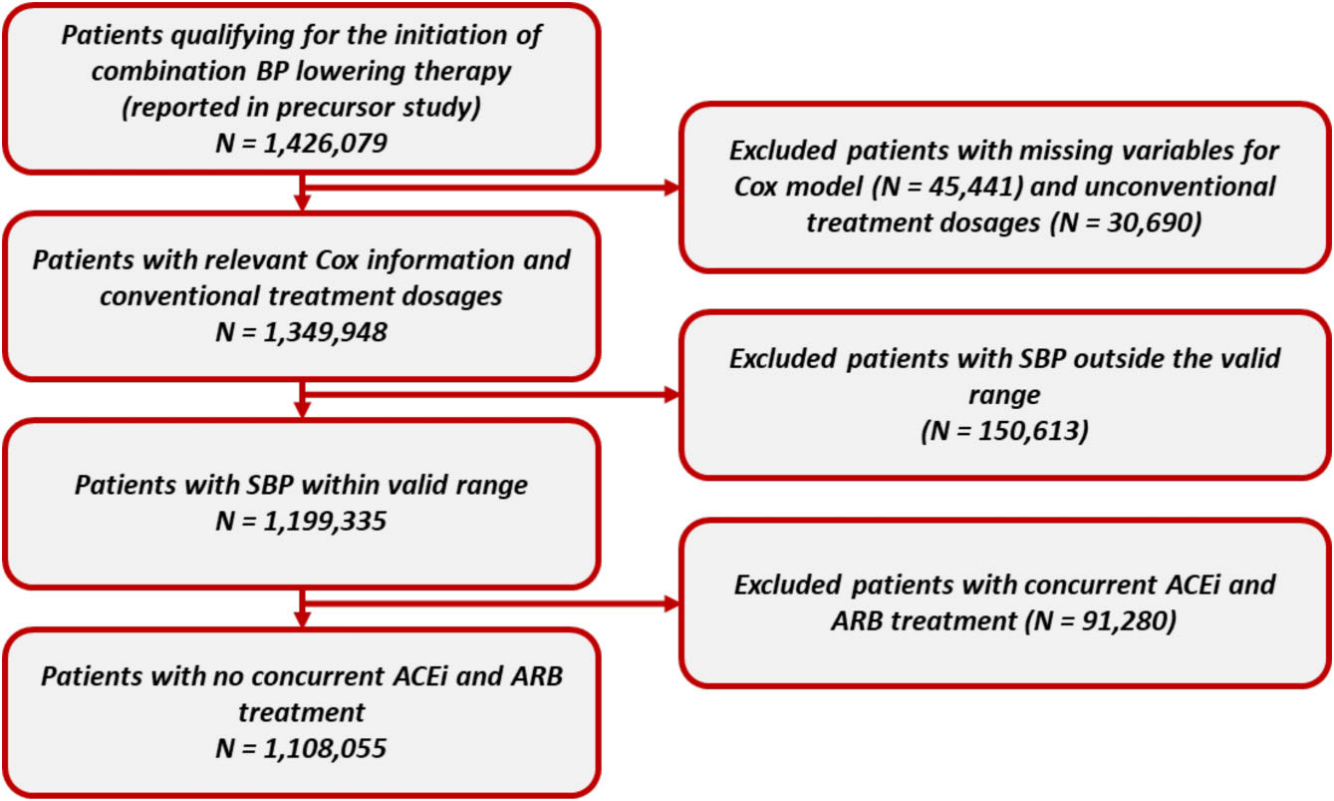
Consort diagram for patient selection. ACEi, angiotensin converting enzyme inhibitor; ARB, angiotensin receptor blocker; BP, blood pressure; SBP, systolic blood pressure.

To enable the patient-level simulation, three additional exclusion criteria were applied, as seen in *Figure 1*. First, patients were excluded if information required to estimate patient-specific baseline risk was not available. Secondly, patients were excluded if their reported dose was deemed ‘unconventional’. Determination of unconventional dosage is explained in Supplementary material online, *Methods*. Additionally, upon estimation of each patient's baseline clinic SBP, i.e. removing ACEi, ARB, and CCB treatment effect, if their SBP value fell outside the acceptable range (90–200 mmHg), as suggested by clinical experts and observed in literature,^13,14^ the patient was excluded. Lastly, patients were excluded if their prescription information indicated concurrent ACEi and ARB usage, as this was deemed unlikely and potentially problematic for some patient risk groups.^15,16^ Subgroups of interest represented patients with or without atherosclerotic cardiovascular disease (ASCVD) or diabetes at baseline.

### Primary endpoint and baseline risk

The primary endpoint represented a composite of non-fatal myocardial infarction (MI), non-fatal stroke (ischaemic or haemorrhagic), non-fatal heart failure hospitalization, or CV death. The methodology for identification of the primary endpoint in the database has been described previously.^9^ The Cox model described in the previous study provided a mechanism for estimating the patient-level observed risk of the primary endpoint. This observed risk was then converted to ‘untreated’ risk (referred to as baseline risk in the subsequent discussion), defined as the expected risk without antihypertensive therapy. This estimation was required as a starting point in order to ensure internal consistency of the simulation. Details on estimation of baseline risk are provided in Supplementary material online, *Methods*.

### Modification of risk with antihypertensive therapy

The BPLTTC meta-analysis^5^ provides a mechanism for relating magnitude of clinic SBP lowering ($\Delta {\mathrm{SBP}}\!\! $) from antihypertensive therapy to the expected reduction in CV event risk. The BPLTTC meta-analysis was based on individual patient-level data from 48 RCTs representing 344 716 participants and concluded that a $\Delta {\mathrm{SBP}}$ of 5 mmHg resulted in a relative risk reduction (RRR) for major CV events of ∼10% at 4 years. This RRR stayed largely consistent across baseline conditions such as ASCVD status and baseline SBP levels.

For each patient in the simulation, $\Delta {\mathrm{SBP}}$ was estimated for the given antihypertensive therapy and represented a patient-specific measure. This estimate was obtained via a random sampling from the reported distributions of treatment-specific $\Delta {\mathrm{SBP}}$ in Law *et al*.,^10^ available in Supplementary material online, *Table S1*. Applying the approach described in Cannon *et al*.,^17^ a log-linear model was assumed for relating $\Delta {\mathrm{SBP}}$ to risk modification. The interpretation of the parameter $\alpha $ is the RRR per 1 mmHg of $\Delta $SBP. Further details are provided in Supplementary material online, *Methods*.

### Monte Carlo simulation

Monte Carlo simulation refers to a generic methodology for generating population-level estimates of an outcome via detailed and probabilistic modelling of the intervention and response at an individual level. The approach was similar to previous reports implementing Monte Carlo simulation on databases, mainly in the area of lipid-lowering therapy.^17,18^  *Figure 1* summarizes the approach for the current investigation. In a typical Monte Carlo analysis, the simulation sample size is identical to the size of the study population. However, based on runtime considerations, we chose a random sample of *N* = 100 000 from the study population for each simulation iteration. Each sampled patient cycled over a 1-day interval, and a CV event or death was probabilistically generated in accordance with the background hazard (instantaneous probability) of these events at that time. The key outcome measure of the simulation was the absolute risk reduction (ARR), which was defined as the difference in event rates for patients receiving a particular treatment strategy relative to the specified reference group. The Monte Carlo simulation was programmed in Python 3.9.^19^

### Scenarios and outcome measures

Five scenarios corresponding to different status with antihypertensive therapies were investigated: (i) untreated, meaning no antihypertensive therapy; (ii) monotherapy with Irbesartan (I) with 100% persistence; (iii) dual therapy with irbesartan + amlodipine (I + A) with 100% persistence; (iv) dual therapy with I + A with 50% persistence; and (v) observed clinical practice. Scenario 3 represents optimal treatment with contemporary guideline recommendations and Scenario 4 represents a suboptimal version of Scenario 3. Scenario 2 representing monotherapy with 100% persistence was included to facilitate comparison with dual therapy.

Contemporary evaluation of patterns of persistence with antihypertensive drug therapies, defined as the act of continuing with treatment (there is some inconsistency in literature on the terminology concerning adherence and persistence),^20–22^ indicates that persistence at 1 year is ∼50%, meaning 50% discontinue by that time since initiation.^20,21,23–25^ This informed the assumptions for Scenario 4, which considered only suboptimal persistence. The detailed logic was such that 50% discontinued by year 1 based on a constant hazard model, and after that patients were still able to discontinue according to the same constant hazard model. It did not consider adherence, which is defined as the act of taking medications in concordance with prescribed regimen, conditional on not discontinuing, for example, an absence of short-term interruptions.^20–22^ Though both are important, among adherence and persistence, the latter is more amenable to an objective estimation from databases, and is also a measure that has been utilized in comparison of dual antihypertensive therapy with FDC and SPC.

Dual therapy in Scenarios 3 and 4 represented irbesartan 150 mg + amlodipine 5 mg, and monotherapy in Scenario 2 represented irbesartan 150 mg. We replicated these scenarios with dual therapy representing ramipril 5 mg + amlodipine 5 mg and monotherapy representing ramipril 5 mg and amlodipine 5 mg, separately. These agents are of interest as, according to a recent commentary on the ideal characteristics of SPCs, ramipril with amlodipine and irbesartan with amlodipine are among the ‘preferred’ agents for the treatment of hypertension and ‘preferred’ class combinations.^26^ Dipette *et al*.^26^ described the combinations most often recommended by clinical guidelines as renin–angiotensin–aldosterone system inhibitors combined with CCB. Furthermore, Law *et al*^10^ analysed 354 RCTs to assert the BP-lowering efficacy by drug class. This evidence was used in our analysis to infer the BP-lowering effect of irbesartan, ramipril, and amlodipine, suggesting the results are applicable and generalizable to other drugs within the ACEi and ARBs classes. All scenarios were replicated for key subgroups.

## Results

A total of 1 108 055 patients met the selection criteria (*Figure 1*). The mean (SD) age was 61.9 (14.0) years and ∼51% were male (*Table 1*). The most prevalent comorbidities were diabetes (13.7%), followed by coronary heart disease (10.8%), chronic kidney disease (CKD; Stage III–V) (9.8%), cerebrovascular disease (4.3%), atrial fibrillation (4.0%), and peripheral arterial disease (2.9%). The subgroup with ASCVD (*N* = 172 722) was older and more likely to have CV and non-CV-related comorbidities compared with the subgroup without ASCVD (*N* = 935 333). The subgroup with diabetes (*N* = 152 666) was also more comorbid compared with the subgroup without diabetes (*N* = 955 389), though to a lesser extent than those with ASCVD.

**Table 1:** Baseline characteristics: overall population and atherosclerotic cardiovascular disease or diabetes subgroups

		Overall (*N* = 1 108 055)	ASCVD (*N* = 172 722)	No ASCVD (*N* = 935 333)	Diabetes (*N* = 152 666)	No Diabetes (*N* = 955 389)
Year of inclusion in study (%)	2005–2009	32.2	31.0	32.6	26.5	33.3
	2010–2014	30.6	30.9	30.5	30.8	30.6
	2015–2019	37.1	38.1	36.9	42.7	36.2
**Demographics (%)**						
Age (years)	Mean (SD)	61.9 (14.0)	71.0 (12.4)	60.2 (13.6)	63.7 (13.7)	61.6 (14.0)
Sex (%)	Male	51.1	58.0	49.8	56.8	50.2
Smoking status (%)	Current smoker	27.4	29.2	27.0	27.7	27.3
	Ex-smoker	37.4	45.2	36.0	43.5	36.5
	Non-smoker	35.2	25.6	36.9	28.9	36.2
**CV-related comorbidities (%)**						
History of CHD	Prior MI	3.6	23.2	0.0	6.7	3.1
	CHD w/o prior MI	7.2	46.5	0.0	11.7	6.5
History of CBVD	Prior stroke	3.8	24.1	0.0	5.4	3.5
	CBVD w/o prior stroke	0.5	3.4	0.0	0.8	0.5
PAD	Yes	2.9	18.7	0.0	5.7	2.5
Heart failure	Yes	2.6	11.1	1.1	5.3	2.2
Atrial fibrillation	Yes	4.0	12.2	2.5	5.3	3.8
**Other comorbidities (%)**						
CKD	Stage ≤II	90.2	77.6	92.5	81.8	91.4
	Stage III	8.2	17.7	6.5	14.2	7.3
	Stage IV	0.5	1.2	0.3	1.2	0.4
	Stage V	1.1	3.5	0.7	2.8	0.9
**Baseline clinic SBP and DBP (%)**						
SBP (mmHg)	140–149	28.4	40.4	26.2	39.8	26.6
	150–159	29.3	28.0	29.5	30.1	29.2
	160–169	21.2	17.1	22.0	17.5	21.8
	≥170	21.1	14.5	22.3	12.6	22.4
DBP (mmHg)	<70	3.7	8.9	2.8	7.3	3.1
	70–79	13.1	23.7	11.1	21.2	11.8
	80–89	30.0	37.1	28.7	36.0	29.1
	90–99	30.1	20.8	31.8	24.4	31.0
	100–109	17.4	7.5	19.2	9.0	18.7
	≥110	5.7	2.0	6.4	2.2	6.3
**Baseline treatments**						
ACEi	Yes	17.8	19.5	17.5	33.7	15.3
ARB	Yes	4.1	5.1	3.9	7.5	3.6
CCB	Yes	14.1	14.6	14.0	10.9	14.6
Thiazide diuretics	Yes	6.3	8.2	6.0	5.7	6.4
β-Blockers	Yes	7.6	20.9	5.1	8.1	7.5
Other diuretics^a^	Yes	0.1	0.3	0.1	0.2	0.1
α-Blockers	Yes	0.5	0.7	0.5	0.8	0.5
LLT	Yes	25.6	58.7	19.5	57.6	20.5
Anticoagulants	Yes	2.8	8.6	1.8	4.0	2.6
Antiplatelets	Yes	13.7	51.9	6.6	26.6	11.6

ACEi, angiotensin-converting enzyme inhibitor; ASCVD, atherosclerotic cardiovascular disease; ARB, angiotensin receptor blocker; CBVD, cerebrovascular disease; CCB, calcium channel blocker; CHD, coronary heart disease; CKD, chronic kidney disease; COPD, chronic obstructive pulmonary disease; CV, cardiovascular; DBP, diastolic blood pressure; LLT, lipid-lowering therapy; MI, myocardial infarction; PAD, peripheral arterial disease; SBP, systolic blood pressure; SD, standard deviation.

^a^Other diuretics include spironolactone, eplerenone, triamterene, and amiloride.

In the overall population, the estimated 10-year event rate for the primary endpoint for the untreated scenario was 22.4%. The estimated 10-year event rates for monotherapy with 100% persistence were 17.0%, 17.6%, and 17.6% for irbesartan (I), ramipril (R), and amlodipine (A), respectively (see Supplementary material online, *Figure S1*). These rates were only modestly better than that observed in clinical practice 17.8%. In Scenario 3 representing 100% persistence to dual therapy, the estimated 10-year event rates for the primary endpoint were 13.6% (ARR = 8.7% compared with untreated) and 14.3% (ARR = 8.0% compared with untreated) for combinations of irbesartan and amlodipine (I + A) and ramipril and amlodipine (R + A), respectively. By comparison, in Scenario 4 representing 50% persistence to dual therapy, the estimated 10-year event rates for the primary endpoint were close to those in the untreated scenario (ARR = 0.2% and 0.1%, respectively, for I + A and R + A compared with untreated).

Among the subgroups, the 10-year event rate for the primary endpoint as observed in clinical practice was highest for those with ASCVD (42.7%), which was nearly 3.2 times higher than for those without ASCVD (13.3%). Similarly, the observed event rate for the group with diabetes was >1.8 times higher than for those without diabetes (28.3% and 16.1%, respectively). For Scenario 3 representing 100% persistence with dual therapy of I + A, the absolute risk of the primary endpoint was reduced by 15.9% and 6.6% in those with and without ASCVD, respectively (see Supplementary material online, *Table S2*). Similarly, the absolute risk was reduced by 11.7% and 7.8% in those with and without diabetes, respectively. In Scenario 4 representing 50% persistence with dual therapy, the ARR relative to the untreated subgroup was 0.6% and 0.8% for the subgroups with ASCVD or diabetes, respectively, for both I + A and R + A, indicating a net clinical benefit of nearly zero with suboptimal persistence to dual therapy.

**Figure 2:**
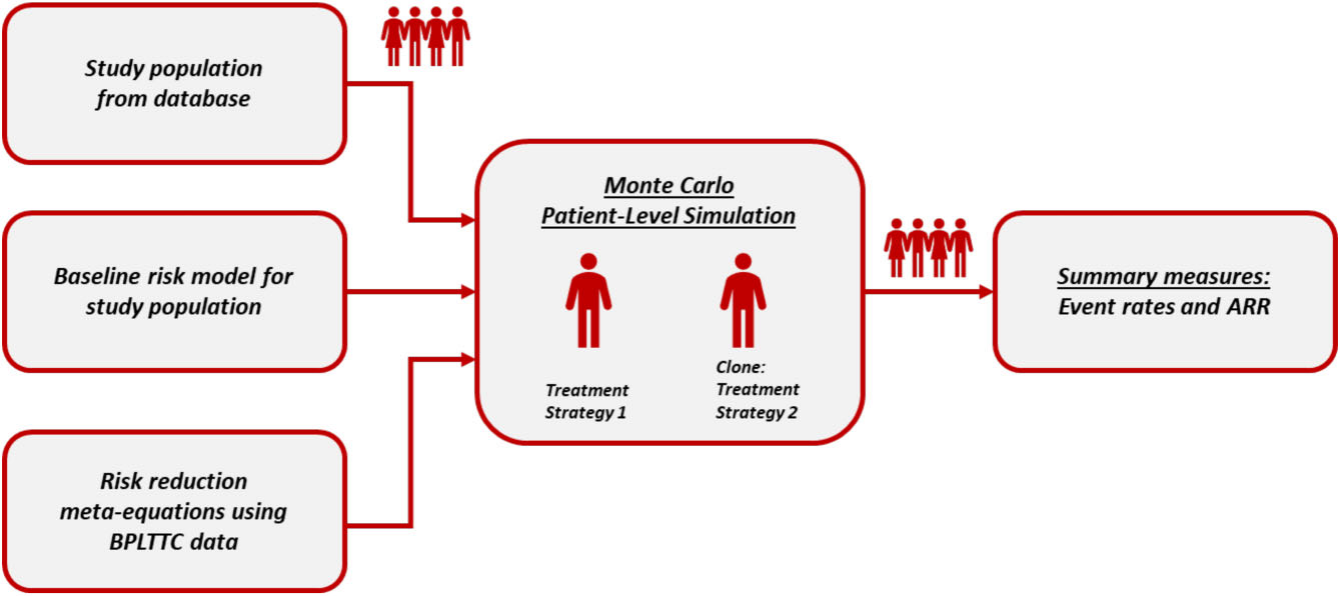
Schematic of the Monte Carlo simulation. ARR, absolute risk reduction; BPLTTC, Blood Pressure Lowering Treatment Trialists Collaboration.

**Figure 3:**
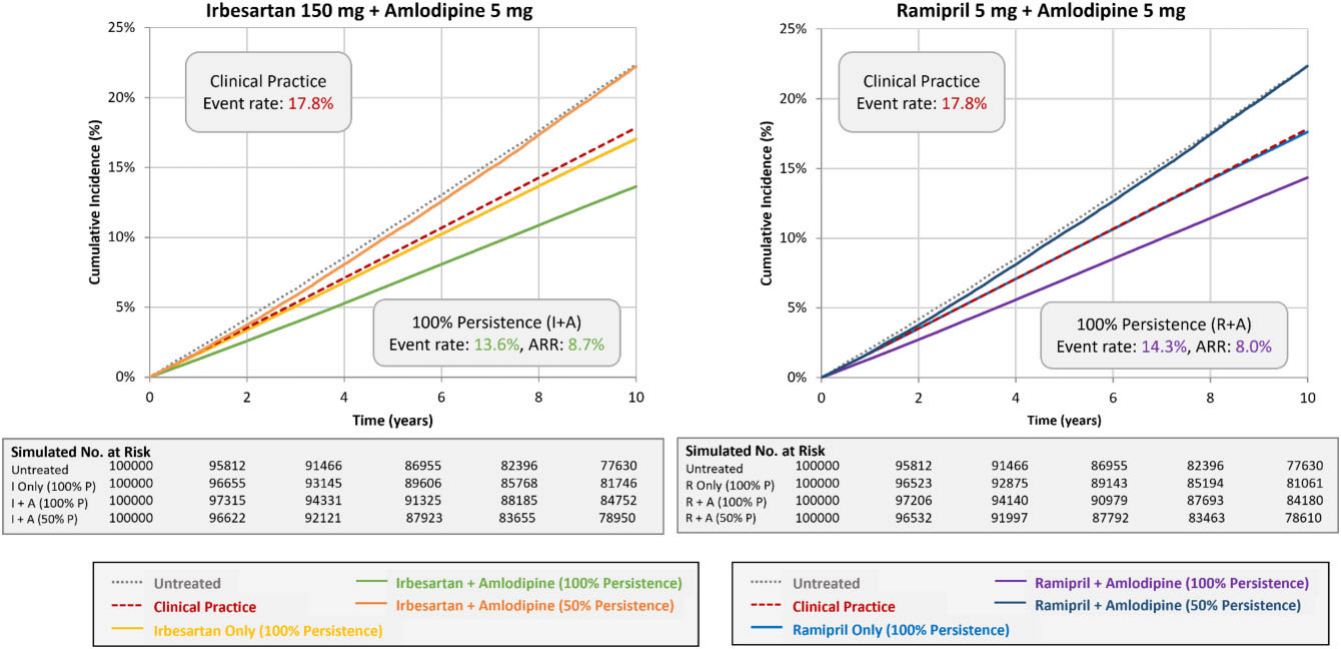
Kaplan–Meier event rates for the primary endpoint: overall population. A, amlodipine; ARR, absolute risk reduction; I, irbesartan; P, persistence; R, ramipril.

**Figure 4:**
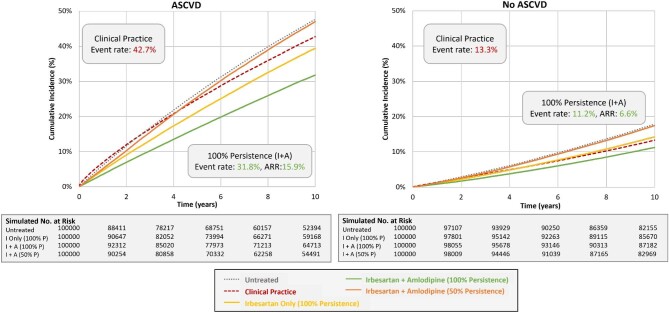
Kaplan–Meier event rates for the primary endpoint for strategies with irbesartan and amlodipine: atherosclerotic cardiovascular disease subgroups. A, amlodipine; ARR, absolute risk reduction; ASCVD, atherosclerotic cardiovascular disease; I, irbesartan; P, persistence; R, ramipril.

**Figure 5:**
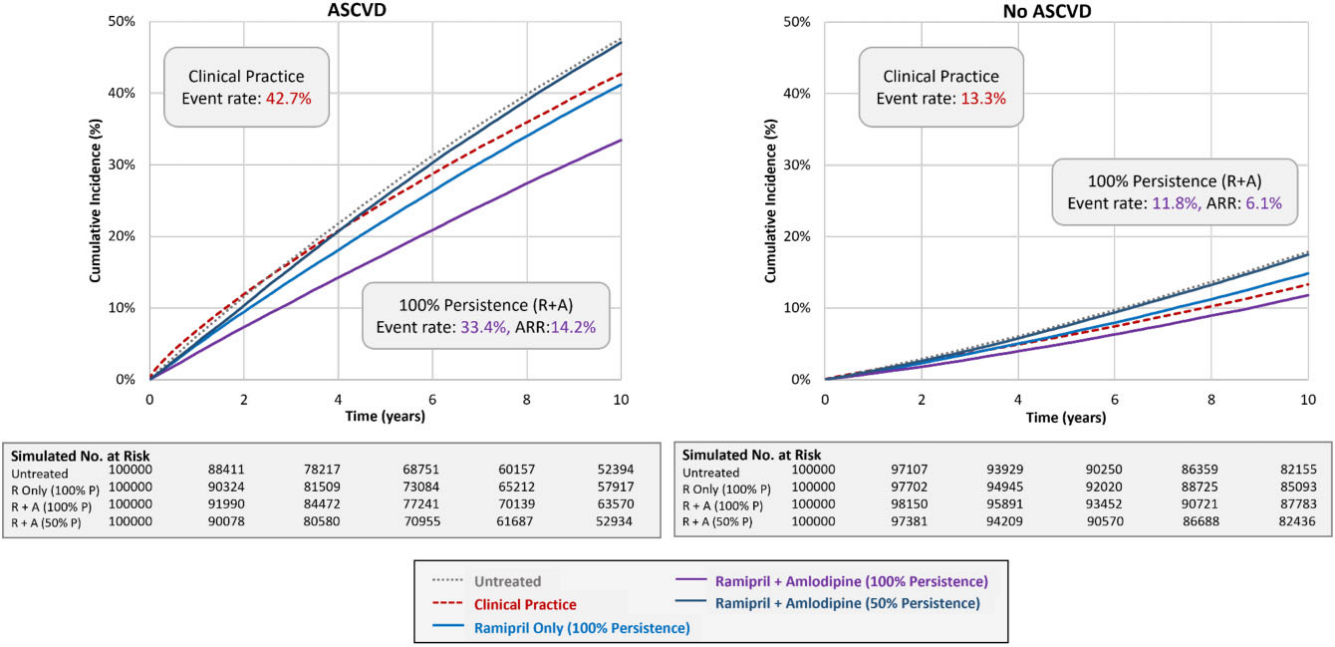
Kaplan–Meier event rates for the primary endpoint for strategies with ramipril and amlodipine: atherosclerotic cardiovascular disease subgroups. A, amlodipine; ARR, absolute risk reduction; ASCVD; atherosclerotic cardiovascular disease; I, irbesartan; P, persistence; R, ramipril.

**Figure 6:**
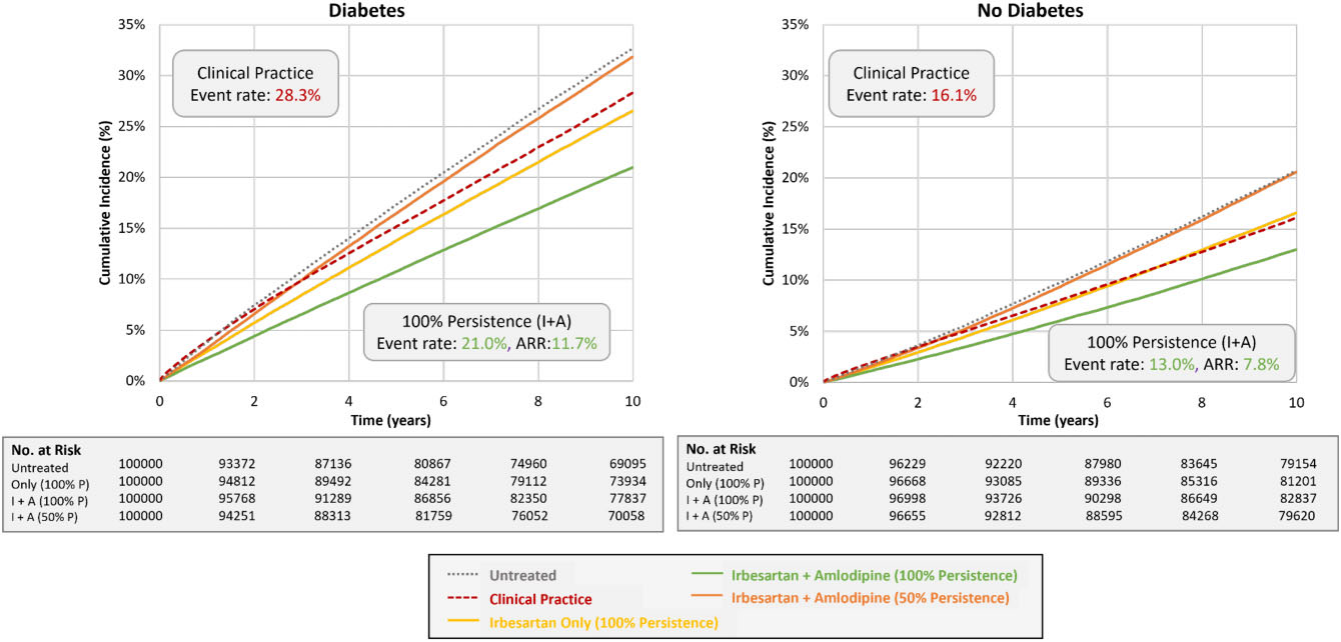
Kaplan–Meier event rates for the primary endpoint for strategies with irbesartan and amlodipine: diabetes subgroups. A, amlodipine; ARR, absolute risk reduction; I, irbesartan; P, persistence; R, ramipril.

Supplementary material online, *Table S2*, and *Figures 2*, *3*, and *5* summarize the findings for the overall population and subgroups based on ASCVD or diabetes status for I + A. Supplementary material online, *Table S2*, and *Figures 2*, *4*, and *6* summarize these same findings for strategies based on R + A. Supplementary material online, *Figures S1**–S5*, and Supplementary material online, *Table S2*, summarize the results for amlodipine monotherapy.

**Figure 7:**
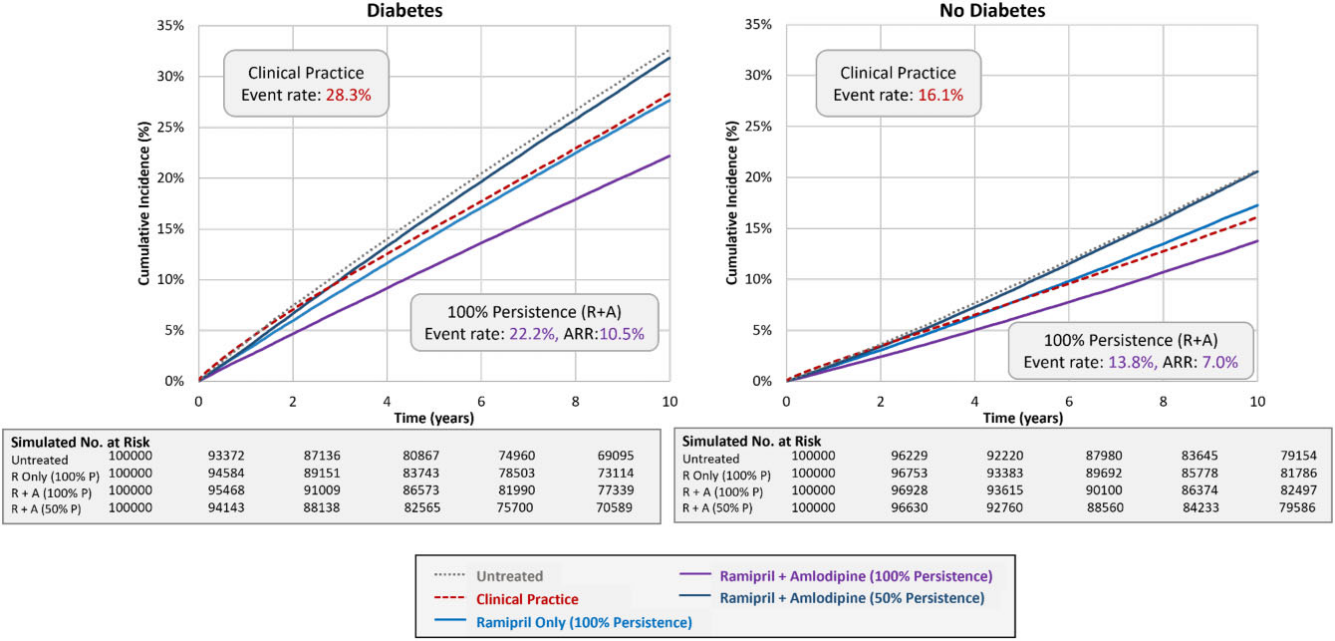
Kaplan–Meier event rates for the primary endpoint for strategies with ramipril and amlodipine: diabetes subgroups. A, amlodipine; ARR, absolute risk reduction; I, irbesartan; P, persistence; R, ramipril.

## Discussion

Elevated BP is a major modifiable CV risk factor, the lowering of which is an evidence-based strategy for reduction of CV risk in a range of at-risk populations.^1–5^ A recent study from the global CV risk consortium^27^ evaluated five modifiable risk factors for CV disease (body mass index, SBP, low-density lipoprotein cholesterol, smoking, and diabetes) from individual-level data representing 1.5 million participants in 34 countries. Among them, elevated BP had the highest population attributable fraction for CV disease at 29.3% and 21.6% among women and men, respectively. Overall, this indicates that optimal control of hypertension offers the greatest potential for reduction of CV events at a population level. Using a Monte Carlo simulation applied to a large population qualifying for initiation of dual antihypertensive therapy, we provide insights as to how narrowing the gaps in clinical practice relative to guideline recommendation could considerably lower the incidence of major CV events.

We summarized the magnitude of clinical benefit with a given strategy via ARR, which measures the difference in expected 10-year event rates with a given treatment strategy vs. a comparator. The ARR, which is regarded as one of the most relevant clinical effect measures, can also be regarded as multiplication of the 10-year event rate of the comparator strategy and the RRR, with the latter being a relative effect measure of the given strategy vs. the comparator. The clinical practice guidelines and the 2021 BPLTTC meta-analysis note a lack of heterogeneity in the relative treatment effect with BP-lowering therapy.^1–5^ In particular, the RRR per 1 mmHg reduction in SBP remains relatively consistent across patient conditions such as ASCVD, diabetes, CKD, age, sex, and ethnicity. A recent analysis of the Systolic Blood Pressure Intervention Trial (SPRINT) trial also confirmed a lack of heterogeneity of the relative treatment effect across different patient conditions.^28^ In summary, this indicates that nearly all at-risk populations benefit from intensive BP-lowering therapy and the main driver of the magnitude the clinical benefit is the 10-year baseline risk, which is strongly dependent on patient conditions. The recent analysis of the SPRINT trial confirms these points and illustrates that the strategy for selecting patients who derive highest clinical benefit can be largely based on the predicted 10-year CV risk at baseline derived from the patient's clinical presentation at the point of initiation of antihypertensive therapy.^28^ Our analyses provide a structured quantification of these aspects in a different population representing a usual clinical practice setting, across a range of guidelines-based strategies and subgroups. Consistent with the idea of the clinical benefit becoming higher with increasing baseline CV risk, we found that the ARR was nearly double in high-risk subgroups with established ASCVD, or diabetes, as compared in groups without these conditions.

Clinical practice guidelines for the management of arterial hypertension recommend upfront initiation of dual therapy, preferably as SPC, in most patients requiring antihypertensive agents.^1–6^ Thus, the most relevant scenarios in our study are 3 and 4, which investigated dual therapy (I + A) with full and reduced persistence, respectively. In particular, the latter scenario assumed 50% persistence at 1 year, an assumption that is well supported by evidence from large populations representing contemporary clinical practice.^20,21,23–25^ A somewhat surprising finding from our study was that the 10-year event rates with reduced persistence with dual therapy were nearly equivalent to the untreated scenario, meaning that any clinical benefit as measured by the ARR was absent. We have not presented the data for monotherapy with reduced persistence (50%), as clearly this will deliver no net clinical benefit as well. Summary findings from Scenarios 3 and 4 reinforce and quantify a foundational principle in achieving a good clinical outcome with antihypertensive therapy, which is to ensure adherence (i.e. following the prescribed regimen) and persistence (i.e. staying on therapy). Though we have not explicitly considered reduced adherence in our study in addition to reduced persistence, it is inevitable that similar implications would follow from reduced adherence.

In summary, our study lends confirmation to the principle of poor persistence on its own constituting an underestimated and major modifiable risk factor.^20^ General barriers to adherence and persistence can be categorized as sociodemographic, healthcare professional and system-related, therapy-related, condition-related, and patient-related.^20,21^ Though all of these factors are critical and interconnected and can be complex to navigate, therapy-related factors can be more amenable to relatively simple and inexpensive solutions such as simplification of complex regimens via SPC as opposed to FDC when multiple agents are required.^20,21^ In hypertension, a recent meta-analysis confirmed the strategy of SPC as compared with FDC results in improved adherence and persistence.^29^ Evidence also suggests that upfront initiation of SPC independently is associated with improved adherence and persistence as compared with initiation with FDC or monotherapy.^25^ The other benefit of upfront initiation of SPC as compared with monotherapy is the elimination of clinical inertia in terms of the need for uptitration or therapy modification at a later stage.^25^ Other than simplification of regimen such as via SPC, evidence suggests that adherence and persistence can be improved by patient engagement and shared decision-making via the process of understanding the severity and consequences of disease, recognizing the benefits of sustained long-term therapy, and self-home BP monitoring and telemonitoring.^20,21,29^ In fact, medication non-adherence is a major cause of treatment-resistant hypertension.^30^ Trimarco *et al*.^31^ found that therapeutic concordance significantly improves the outcome of antihypertensive treatment in a population of patients with treatment resistant hypertension. Trimarco *et al*.^31^ described ‘concordance’ as the requirements for treatment acceptance and implies that all those involved (e.g. patients and physicians) are in agreement in trying to achieve treatment goals. Initiation of a prescription must be recognized as an initial step in the journey, and continued patient–provider engagement, including evaluation of adherence and persistence, should be an integral part of the process of disease management aiming at achieving optimal BP control according to current recommendations.

Analyses of large populations, including previous study of our analysis,^9^ continue to find that the dominant pattern in contemporary clinical practice is initiation of treatment as monotherapy, and in majority of these patients the monotherapy is not uptitrated, resulting in about half of treated hypertensives having their BP uncontrolled in most countries.^21,23–25^ Clinical inertia, defined as ‘failure of health care providers to initiate or intensify therapy according to current guideline’, remains a challenge in therapy optimization across multiple domains in CV disease prevention, including BP, lipids, and blood glucose management.^32–34^ The issues are again multifactorial and include lack of awareness of guideline updates, lack of familiarity with updated information on new drugs (including efficacy and adverse reaction), time constraints in clinical encounters, time devoted to counselling patients, competing health priorities for the patient, and clinical inertia.^32–34^ Another potential explanation for a large proportion of patients initiating and remaining on monotherapy could be the perception of an increased risk of adverse events (AEs) with intensive BP-lowering therapy.^28^ Based on RCT data, the reduction in risk of major events with intensive strategy with combination agents far exceeds the increase in risk of AEs with a net positive benefit.^5^ Importantly, the risk reduction for major CV events and increased risk for AEs with intensive therapy should not be considered as clinically equivalent trade-offs as the AEs tend to be infrequent, and relatively mild and transient.^28^

The key strength of our study, evident from the results of the targeted literature search, is that it likely represents the first of its kind to apply a rigorous Monte Carlo simulation to investigate the pragmatic population-level implications of following guidelines-based recommendations for dual BP-lowering therapy. Our analysis utilized the largest source of randomized evidence of BP-lowering effects on CV disease and death published by the BPLTTC, ensuring that our applied risk reduction mechanism is valid, applicable for our study population, and reflects the most contemporary evidence with regard to similar proportional effects in people with or without previousCV disease and across categories of baseline SBP. Our analysis also provides evidence on comparison of dual combination strategies with ACEi and ARBs together with CCBs, which has only been investigated head-to-head in two clinical trials. An improvement from prior research, our study utilized a large and contemporary population qualifying for BP-lowering therapy, representing usual clinical practice rather than research conditions, with a long follow-up of 10 years. Lastly, the linked nature of databases (CPRD, HES, and ONS) enabled coverage of information across primary care and hospitalization settings, as well as death status, which enhanced the reliability of patient characterization, treatment status and SBP during follow-up, and hard endpoints.

Limitations of our study include those inherent to investigations based on observational data, such as ascertainment of treatment status from prescription records, coding accuracy, and potentially incomplete recording of certain data. The ascertainment of treatment status is limited to fill and run-out dates of prescriptions, which does not accurately reflect patients actually taking the medication they receive. In that sense, our analysis may reflect an overestimation of the untreated event risk given we removed the effect of treatments under the assumption that patients had been 100% persistent and adherent to all observed prescriptions. At the same time, however, there may be incomplete recording of data, including prescriptions and baseline medical history, that would alter our risk predictions in terms of missing information on patient treatment status and baseline risk. Other limitations include the modelling assumptions that were required to operationalize the simulation, including the estimation of the baseline risk and modification of risk with BP lowering. It is possible that other risk factors or model forms may be used to estimate the baseline risk than what we have reported in the Supplementary material online, *Methods* section, resulting in varied findings. Our analysis was also limited to three classes of agents, namely ACEi, ARBs, and CCBs, which are currently recommended as first-line treatment for hypertension by most guidelines, and in these we only focused on ramipril, irbesartan, and amlodipine, which are widely used globally. Other classes such as diuretics and ${\mathrm{\beta }}$-blockers, and other agents, play a key role in the management of hypertension, and future analyses should be conducted to investigate other drugs within these classes and alternate class combinations. Additional future analyses of interest include assessing what proportion of patients achieve their target BP and whether there is any difference regarding target BP achievement in any group. This would enable providers to further identify other target subgroups, for example those with a history of heart failure, requiring intensification of treatment or needing encouragement to persist and adhere to prescribed treatments. Non-pharmacological options, such as modification of diet, lifestyle, and exercise, are also crucial in comprehensive management of hypertension, and were not considered in our study. Finally, an explanation for the suboptimal observed clinical practice in our data could be due to the study population's representation of individuals in the UK during years 2005–2019, which largely precedes the release of the 2018 ESC/ESH guideline. In addition, contemporary UK NICE guidelines recommend initial treatment with monotherapy in most patients requiring BP-lowering therapy.^6^

## Conclusions

Our simulation analysis implemented on 1.1 million individuals with hypertension indicates substantial opportunity for CV risk reduction by ensuring sustained treatment according to current guideline recommendations. Populations with conditions that prognosticate a higher risk at the point of therapy initiation, such as those with ASCVD or diabetes, derive a multifold higher clinical benefit from guidelines-based therapy. Monotherapy initiation and maintenance continues to be the dominant pattern in observed clinical practice, whereas in many cases initiation of dual therapy would nearly double the clinical benefit compared with monotherapy. Suboptimal persistence has a dominant impact on diminishing the clinical benefit, cementing its stand-alone role as a modifiable risk factor. Overall, this study validates the summary recommendation from major clinical guidelines that advocate upfront initiation of dual therapy, preferably via SPC, and ensuring persistence with therapy over time.

## Supplementary Material

pvae048_Supplemental_File

## Notes

**Conflict of interest:** A.C. has received consulting fees from Sanofi and Menarini; honoraria or other payments for participation in speakers’ bureaus from Berlin-Chemie, Biolab, Ferrer, Menarini, and Sanofi; and honoraria or other payments for participation in advisory boards of Ferrer and Sanofi. C.B. has received consulting fees from Alfasigma, Sanofi, AstraZeneca, Gilead, and Menarini and honoraria or other payments for participation in speakers’ bureaus from Alfasigma, Berlin-Chemie, Servier, Recordati, and Novo Nordisk. G.S.S. has received consulting fees from AstraZeneca, Menarini, Sanofi, Servier, and Viatris; lecture honoraria from AstraZeneca, Menarini, Sanofi, Servier, and Viatris; and travel support from Menarini. J.B. has received consulting fees from Sanofi and Quantum Genomics; honoraria or other payments for participation in speakers’ bureaus from Hikma, Elkendi, Vivactis, Servier, and Mylan; and travel support from Servier. I.K. and M.A.M. are employees and stockholders of Sanofi. A.K. does not have any disclosures to report.

## Data availability

The data underlying this article will be shared on reasonable request to the corresponding author.
