# Supplementary material for: Estimated impact of guidelines-based initiation of dual antihypertensive therapy on long-term cardiovascular outcomes in 1.1 million individuals
Source: Eur Heart J Cardiovasc Pharmacother. 2024 Jul 9;10(8):697–707. doi: 10.1093/ehjcvp/pvae048 (PMC11724143; doi:10.1093/ehjcvp/pvae048)
Supplement: pvae048_Supplemental_File [file pvae048_supplemental_file.docx]

**Supplemental Materials**

**Estimated impact of guidelines-based initiation of dual antihypertensive therapy on long-term cardiovascular outcomes in 1.1 million individuals.**

Antonio Coca^1^ MD PhD, Claudio Borghi^2^ MD PhD, George S. Stergiou^3^ MD PhD, Irfan Khan^4^ PhD, Alexandra Koumas^5^ BS, Jacques Blacher^6^ MD PhD, Mohamed Abdel-Moneim^7,8^ MD

^1^Department of Medicine, University of Barcelona, Hospital Clínic, Barcelona, Spain; University Abat Oliba CEU, Barcelona, Spain.

^2^University of Bologna, Department of Medicine, Science and Surgery, Bologna, Italy

^3^National and Kapodistrian University of Athens, School of Medicine, Hypertension Center STRIDE-7, Third Department of Medicine, Sotiria Hospital, Athens, Greece

^4^Sanofi, New Jersey, United States of America

^5^Axtria Inc., Berkeley Heights, NJ, USA

^6^Hôpital Hôtel-Dieu, AP-HP, Diagnosis and Therapeutic Center, Université Paris Cité

^7^Sanofi, Dubai, United Arab Emirates

^8^Department of Family Medicine, College of Medicine, University of Sharjah, United Arab Emirates.

**Table of Contents**

**Methods A:** Definition of Unconventional Doses

**Methods B:** PubMed Query for Prior Evidence

**Methods C:** Modeling Probability of an Event in the Simulation

**Table S1:** Efficacies of Antihypertensive Therapies based on Law et al.

**Table S2:** Event Rates and ARR for the Primary Endpoint: Overall Population and ASCVD or Diabetes Subgroups

**Figure S1:** Kaplan-Meier Event Rates for the Primary Endpoint Including Amlodipine Monotherapy: Overall Population

**Figure S2.** Kaplan-Meier Event Rates for the Primary Endpoint for Strategies with Irbesartan and Amlodipine, Including Amlodipine Monotherapy: ASCVD Subgroups

**Figure S3.** Kaplan-Meier Event Rates for the Primary Endpoint for Strategies with Ramipril and Amlodipine, Including Amlodipine Monotherapy: ASCVD Subgroups

**Figure S4.** Kaplan-Meier Event Rates for the Primary Endpoint for Strategies with Irbesartan and Amlodipine, Including Amlodipine Monotherapy: Diabetes Subgroups

**Figure S5.** Kaplan-Meier Event Rates for the Primary Endpoint for Strategies with Ramipril and Amlodipine, Including Amlodipine Monotherapy: Diabetes Subgroups

**References**

This supplemental material has been provided by the authors to give readers additional information about their work.

***Methods A:*** ***Definition of Unconventional Doses***

Determination of standard dose for the three agents included in our model was based on the frequency of their occurrence in the data. Most frequent doses of Ramipril, Irbesartan and Amlodipine being 5 mg, 150 mg, and 5 mg, respectively, which were selected as the standard dose of these agents. Conventional doses were defined as those that fall within an upper and lower limit bound around the standard dose. The upper limit for this purpose was calculated by multiplying the standard dose by 10, whereas the lower limit was calculated by multiplying the standard dose by 1/10. Values falling outside the upper and lower limits were termed unconventional dosages, and patients with prescriptions of unconventional dosages were excluded from the analysis.

***Methods B: PubMed Query for Prior Evidence***

We searched the PubMed database for potential articles investigating the impact of guidelines-based antihypertensive treatment on CV event rates via simulation based approaches.

The query was specified as ("2019"[Date - Publication]: "3000"[Date - Publication])) AND (simulation [Title] OR Monte Carlo [Title]) AND (hypertension [Title] OR blood pressure [Title]) AND (event rates [Title] OR (event rate [Title] OR risk [Title] OR [outcome] OR [outcomes]) AND ((European Society of Cardiology) OR (European Society of Hypertension) OR (guidelines) OR (guideline))).

The query resulted in 1 article. Upon manual review it was deemed to be not relevant for stated aims.

***Methods C: Modeling Probability of an Event in the Simulation***

We utilized results from the previous study,*^1^* including observed KM event rates and risk factors identified via Cox model for the primary endpoint, to construct a fully parametric baseline CV event risk model. For the purpose of this study, baseline CV event risk is defined as the expected risk without antihypertensive therapy. The parametric Cox model represents an intuitive starting point for developing the full baseline risk model as a function of patient characteristics, $X$, and time, $t$. We define the hazard function, $\lambda(t, X)$, as the instantaneous risk of experiencing an event on a given day. Then, $\lambda(t, X)$ can be represented by the multiplication of the baseline hazard (or hazard given reference-level factors or the absence of risk factors), $\lambda_{0}\left( t \right),$and the modification of $\lambda_{0}\left( t \right)$ with patient-level risk factors:

| $\lambda\left( t, X \right)= \lambda_{0}\left( t \right)\exp\left( \sum_{j} X_{j}\beta_{j} \right)$ | 1 |
| --- | --- |

where the summation over $j$ implies a linear combination of present risk factors and their influence on the hazard. To develop a fully parametric specification for use in the simulation model, we assumed the following functional form for $\lambda_{0}\left( t \right)$, as utilized and rationalized in previously conducted studies *^2^*^,^*^3^*:

|  | $\lambda_{0}\left( t \right)=A+B \text{exp}\left( -Ct \right)$ | 2 |
| --- | --- | --- |

where $A$, $B$, and $C$ are model parameters. The resulting baseline hazard function can be expressed as:

|  | $\lambda\left( t, X \right)= \left( A+B \text{exp}\left( -Ct \right) \right)\exp\left( \sum_{j} X_{j} \beta_{j} \right)$ | 3 |
| --- | --- | --- |

As described in previously conducted simulation studies *^2^*^,^*^3^*, risk reduction with treatment can be estimated via the following equation:

| $\lambda\left( t, X, \Delta SBP \right)= \left( A+B \text{exp}\left( -Ct \right) \right)\exp\left( \sum_{j} X_{j}\beta_{j} \right)\left( \ln\left( 1- \alpha\right) \Delta SBP \right)$ | 4 |
| --- | --- |

Where $\alpha$ is derived based on evidence from meta-analysis from the BPLTTC *^4^* representing expected risk reduction per 5 mmHg reduction in SBP, and $\Delta SBP$ represents the magnitude of SBP-lowering with antihypertensive treatment. This approach is consistent with that used in Cannon et al *^2^* where a log-linear model of the form $\ln\left( HR \right)=\ln\left( 1- \alpha\right) \Delta SBP$was employed, and $\alpha$ was estimated as $1-\exp\left( \ln\left( HR \right)/\Delta SBP \right)$ with $HR=0.9$ and $\Delta SBP=5$ mm Hg being substituted in this expression per the BPLTTC meta-analysis. *^4^*

Following from this, estimation of the baseline risk model parameters $A$, $B$, and $C$ was undertaken in the following manner. First, we applied equation 4 to each patient in the overall population and probabilistically generated events over time, conditional on patient characteristics, $X$, time, $t$, and $\Delta SBP$. The resulting events were aggregated at the population-level via the Kaplan-Meier method, and the resulting event rates based on the specified model were compared to observed KM events rates from the precursor study. *^1^* The SciPy. Optimize package in Python 3.0 *^5^* was employed to minimize the error between the model estimated event rates and observed event rates, yielding optimized estimates of $A$, $B$, and $C$ for the primary endpoint.

Baseline risk, representing risk without antihypertensive treatment, was estimated by setting $\alpha=0$ in equation 4. Conversely, modified risk with antihypertensive treatment was estimated by substituting the value of $\alpha$ for the primary endpoint and $\Delta SBP$ conditional on the given treatment history in equation 4.

**Table S​1.** **Efficacies of Antihypertensive Therapies based on Law et al.** *^6^*

| **Drug Class** | **Dosage (relative to 1 standard dose)** | **Mean Systolic Blood Pressure Reduction (mm Hg)** | **95% Confidence Interval**  **(mm Hg)** |
| --- | --- | --- | --- |
| **ACEi** | 0.5 | 6.9 | 6.1, 7.8 |
|  | 1 | 8.5 | 7.9, 9.0 |
|  | 2 | 10 | 9.5, 10.4 |
| **ARB** | 0.5 | 7.8 | 7.1, 8.6 |
|  | 1 | 10.3 | 9.9, 10.8 |
|  | 2 | 12.3 | 11.7, 12.8 |
| **CCB** | 0.5 | 5.9 | 5.2, 6.6 |
|  | 1 | 8.8 | 8.3, 9.2 |
|  | 2 | 11.7 | 11.0, 12.3 |

ACEi, angiotensin converting enzyme inhibitor; ARB, angiotensin receptor blocker; CCB calcium channel blocker

**Table S​2.** **Event Rates and ARR for the Primary Endpoint: Overall Population and ASCVD or Diabetes Subgroups**

|  | **Overall**  **(N=1,108,055)** | **ASCVD**  **(N =** **172,722)** | **No ASCVD**  **(N = 935,333)** | **Diabetes**  **(N = 152,666)** | **No Diabetes**  **(N = 955,389)** |
| --- | --- | --- | --- | --- | --- |
| **Event Rate at 10 Years, %** | | | | | |
| 1. Untreated | 22.4 | 47.6 | 17.8 | 32.6 | 20.7 |
| 2a. Monotherapy (A); 100% Persistence | 17.6 | 41.7 | 14.8 | 26.7 | 17.8 |
| 5. Observed Clinical Practice | 17.8 | 42.7 | 13.3 | 28.3 | 16.1 |
| **ARR at 10 Years, % (Reference: Untreated)** | | | | | |
| 2a. Monotherapy (A); 100% Persistence | 4.8 | 5.9 | 3.0 | 5.9 | 2.9 |
| 5. Observed Clinical Practice | 4.6 | 4.9 | 4.5 | 4.3 | 4.6 |
| **Irbesartan and Amlodipine Scenarios** | | | | | |
| **Event Rate at 10 Years, %** | | | | | |
| 2b. Monotherapy (I); 100% Persistence | 17.0 | 39.4 | 14.2 | 26.5 | 16.6 |
| 3. Dual Therapy (I+A); 100% Persistence | 13.6 | 31.8 | 11.2 | 21.0 | 13.0 |
| 4. Dual Therapy (I+A): 50% Persistence | 22.2 | 47.0 | 17.4 | 31.8 | 20.6 |
| **ARR at 10 Years, % (Reference: Untreated)** | | | | | |
| 2b. Monotherapy (I); 100% Persistence | 5.3 | 8.3 | 3.6 | 6.1 | 4.2 |
| 3. Dual Therapy (I+A); 100% Persistence | 8.7 | 15.9 | 6.6 | 11.7 | 7.8 |
| 4. Dual Therapy (I+A): 50% Persistence | 0.2 | 0.6 | 0.4 | 0.8 | 0.2 |
| **Ramipril and Amlodipine Scenarios** | | | | | |
| **Event Rate at 10 Years, %** | | | | | |
| 2b. Monotherapy (R); 100% Persistence | 17.6 | 41.2 | 14.8 | 27.7 | 17.3 |
| 3. Dual Therapy (R+A); 100% Persistence | 14.3 | 33.4 | 11.8 | 22.2 | 13.8 |
| 4. Dual Therapy (R+A): 50% Persistence | 22.3 | 47.1 | 17.5 | 31.8 | 20.6 |
| **ARR at 10 Years, % (Reference: Untreated)** | | | | | |
| 2b. Monotherapy (R); 100% Persistence | 4.8 | 6.5 | 3.0 | 5.0 | 3.5 |
| 3. Dual Therapy (R+A); 100% Persistence | 8.0 | 14.2 | 6.1 | 10.5 | 7.0 |
| 4. Dual Therapy (R+A): 50% Persistence | 0.1 | 0.6 | 0.4 | 0.8 | 0.2 |

A, amlodipine; ARR, absolute risk reduction; ASCVD, atherosclerotic cardiovascular disease; I, irbesartan; R, ramipril.

**Figure S1. Kaplan-Meier Event Rates for the Primary Endpoint Including Amlodipine Monotherapy: Overall Population**


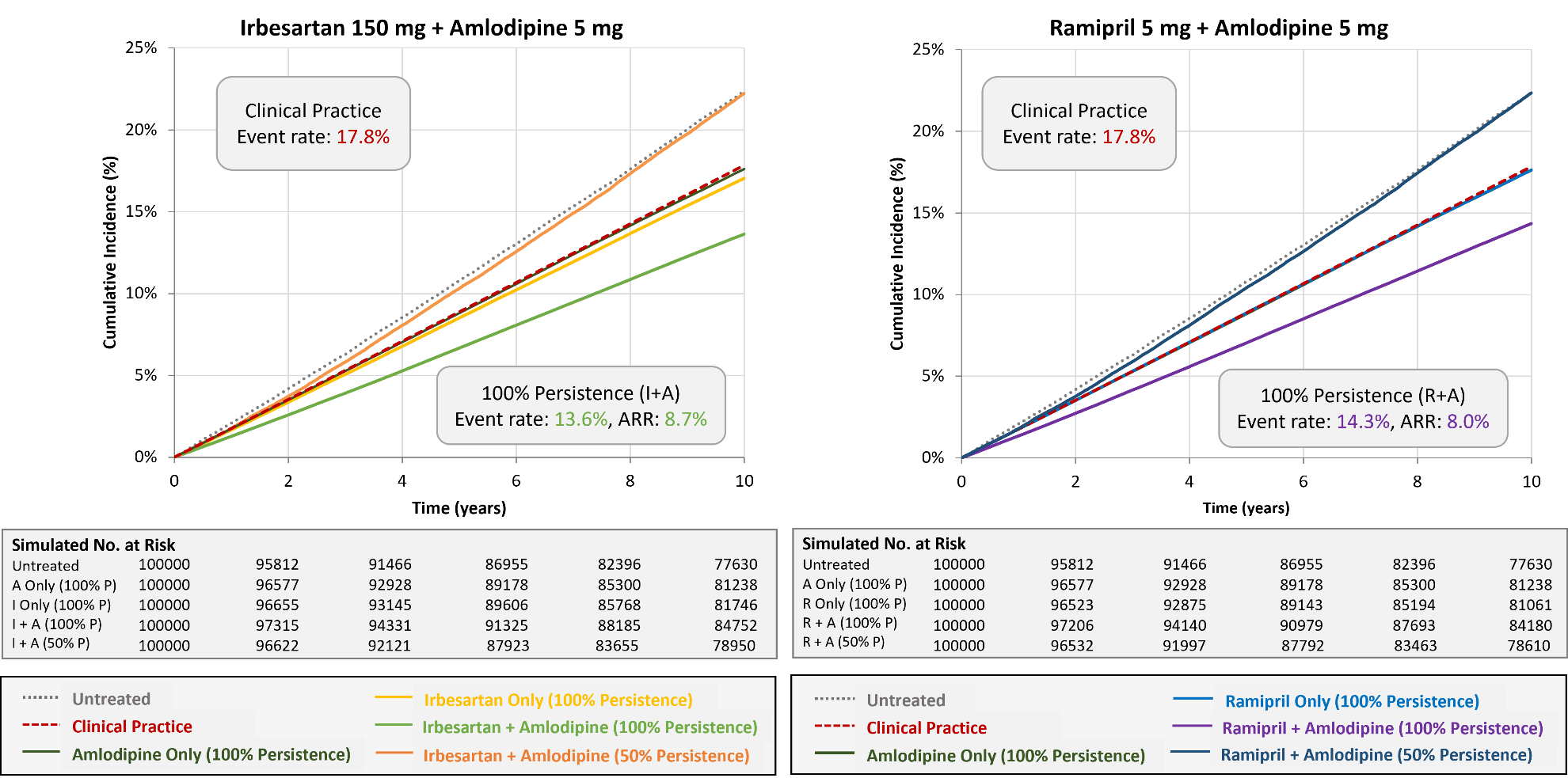


A, amlodipine; ARR, absolute risk reduction; I, irbesartan; P, persistence; R, ramipril.

**Figure S2. Kaplan-Meier Event Rates for the Primary Endpoint for Strategies with Irbesartan and Amlodipine, Including Amlodipine Monotherapy: ASCVD Subgroups**

**
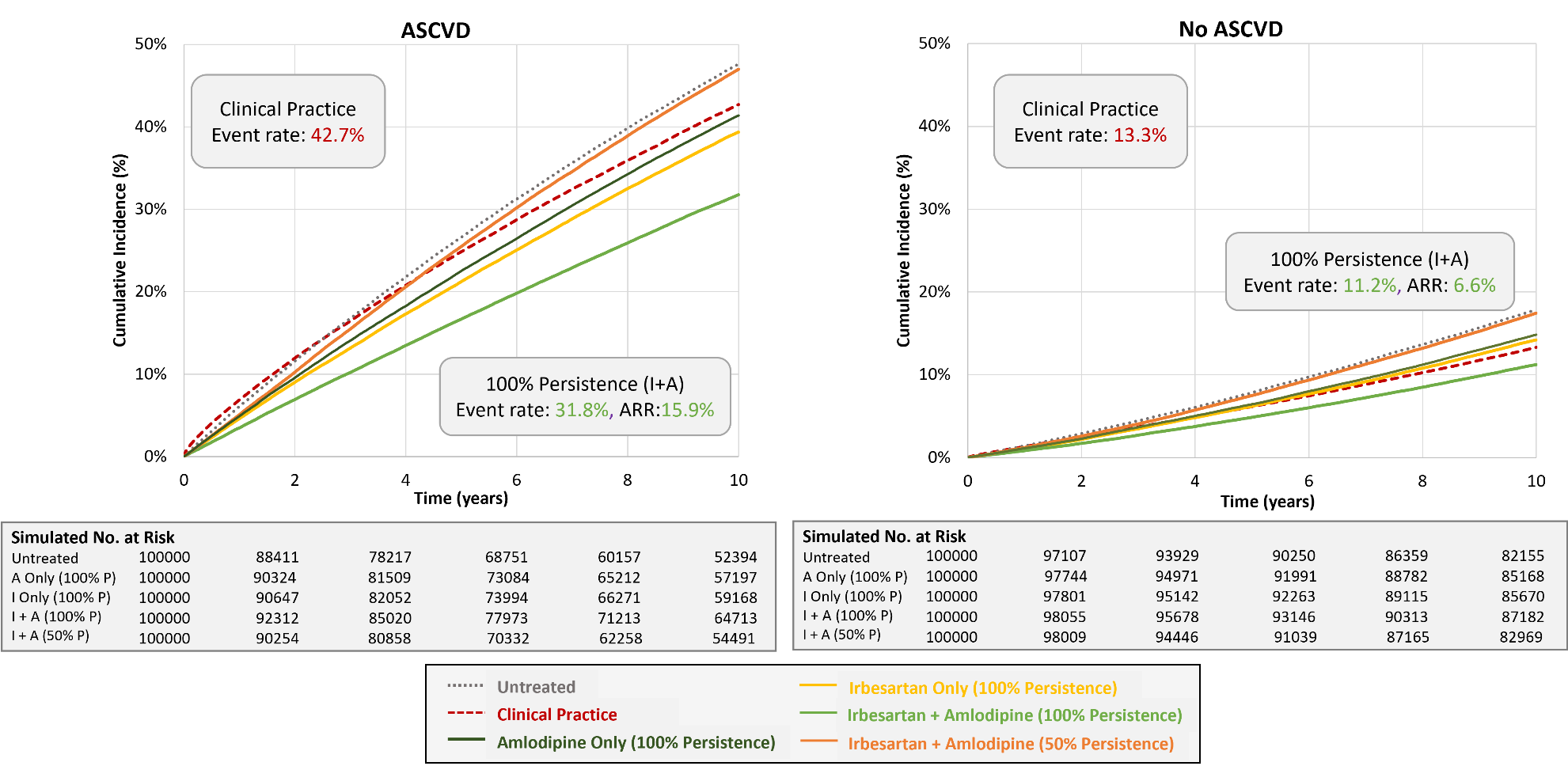
**

A, amlodipine; ARR, absolute risk reduction; I, irbesartan; P, persistence; R, ramipril.

**Figure S3. Kaplan-Meier Event Rates for the Primary Endpoint for Strategies with Ramipril and Amlodipine, Including Amlodipine Monotherapy: ASCVD Subgroups**

**
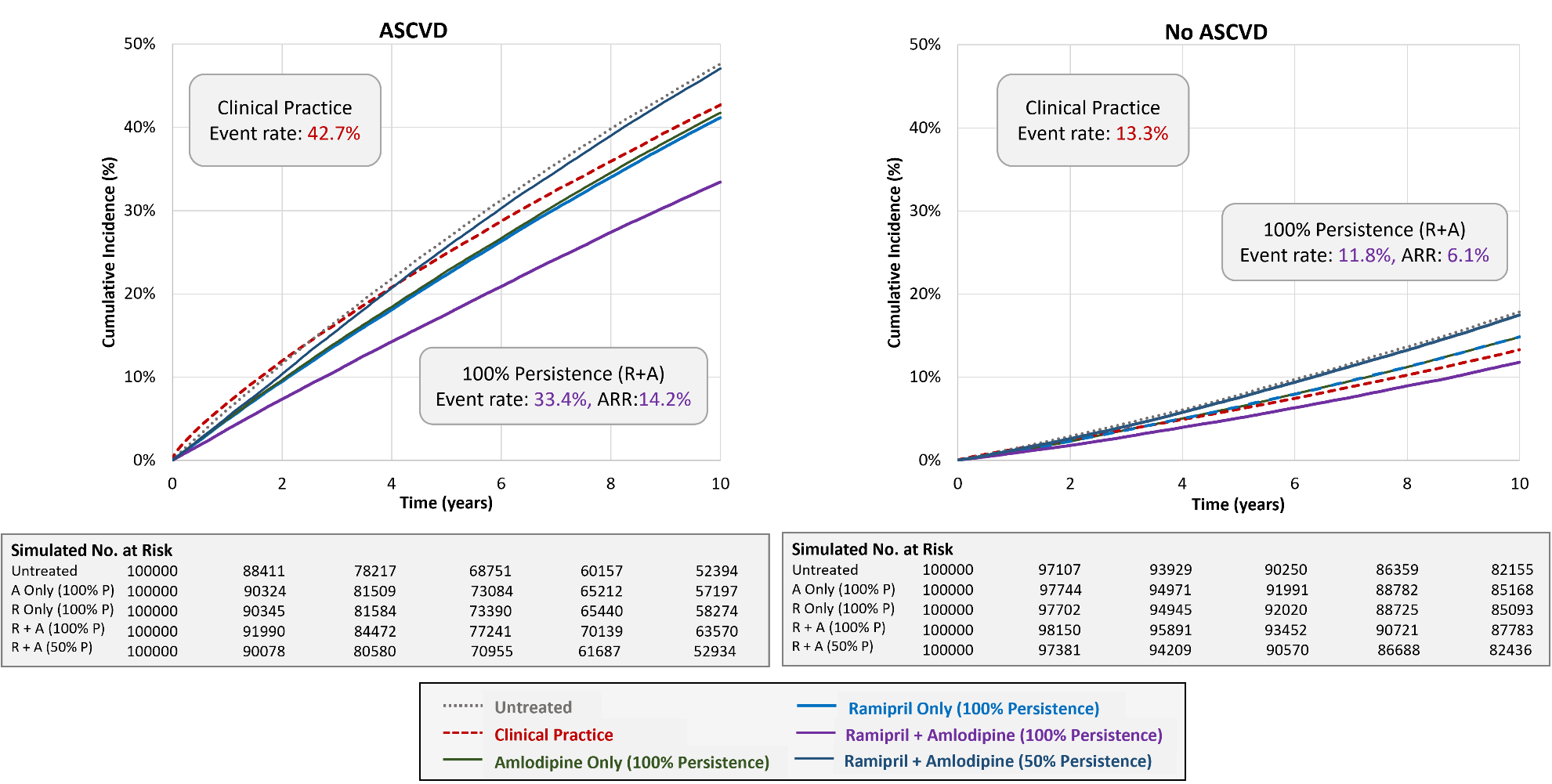
**

A, amlodipine; ARR, absolute risk reduction; I, irbesartan; P, persistence; R, ramipril.

**Figure S4. Kaplan-Meier Event Rates for the Primary Endpoint for Strategies with Irbesartan and Amlodipine, Including Amlodipine Monotherapy: Diabetes Subgroups**

**
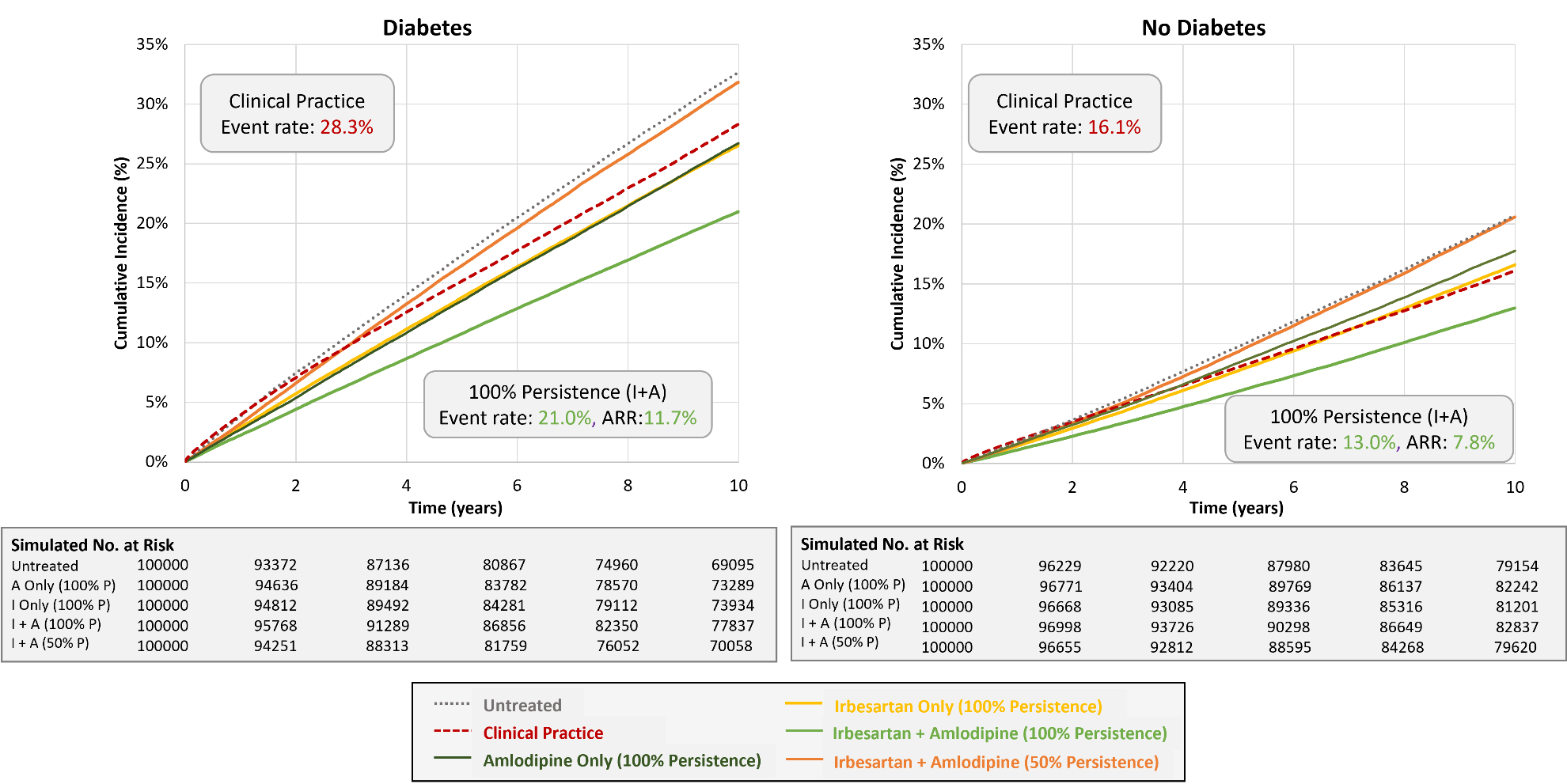
**

A, amlodipine; ARR, absolute risk reduction; I, irbesartan; P, persistence; R, ramipril.

**Figure S5. Kaplan-Meier Event Rates for the Primary Endpoint for Strategies with Ramipril and Amlodipine, Including Amlodipine Monotherapy: Diabetes Subgroups**

**
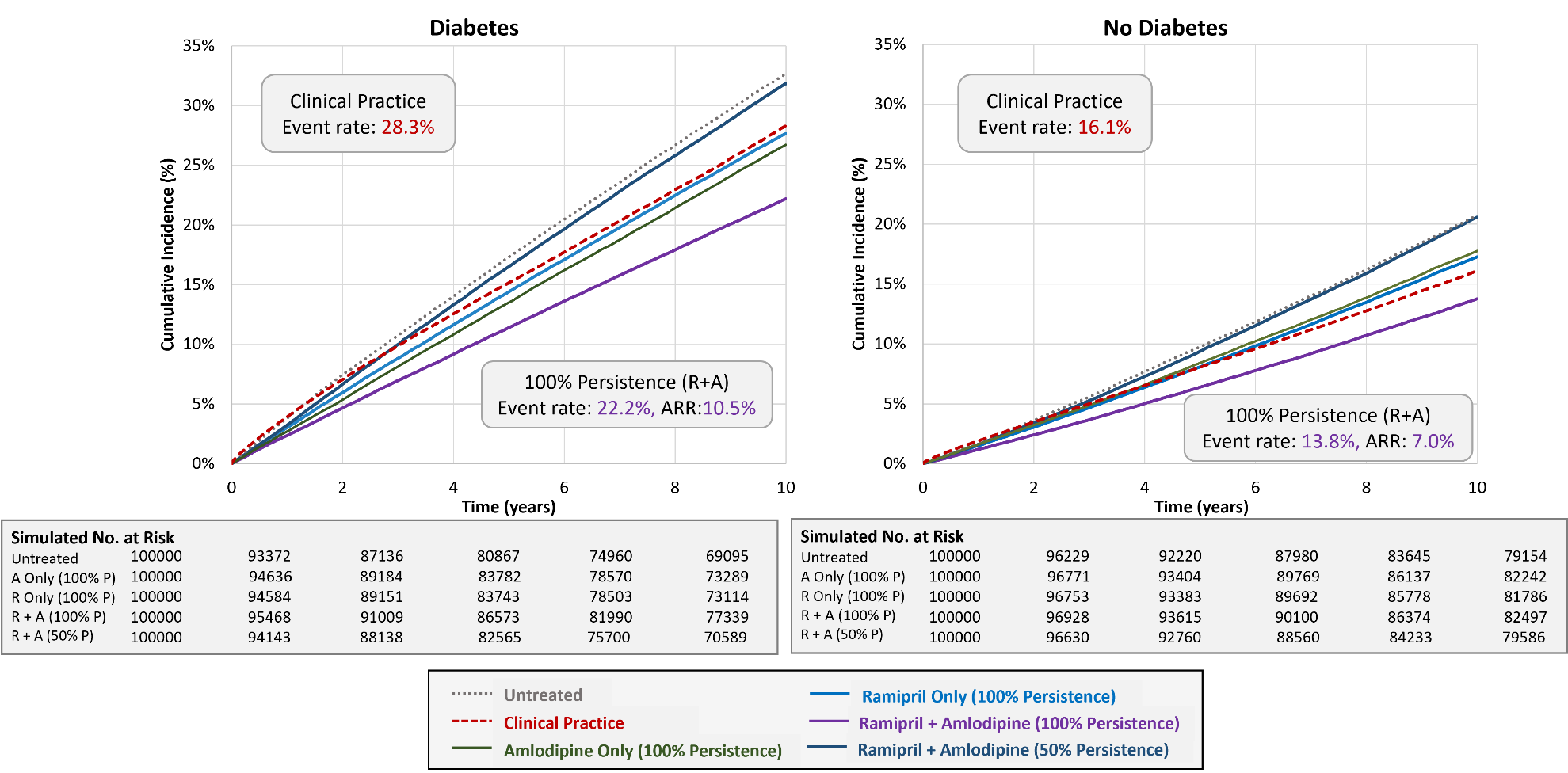
**

A, amlodipine; ARR, absolute risk reduction; I, irbesartan; P, persistence; R, ramipril.

**References**

1. Coca A, Borghi C, Stergiou GS, Blacher J, Lee C, Tricotel A, et al., Long-term event rates, risk factors, and treatment pattern in patients qualifying for dual blood pressure-lowering therapy: an observational study in 1.4 million individuals. J Hypertens 2023; 41: e29-e30.

2. Cannon CP, Khan I, Klimchak AC, Sanchez RJ, Sasiela WJ, Massaro JM, et al., Simulation of impact on cardiovascular events due to lipid-lowering therapy intensification in a population with atherosclerotic cardiovascular disease. Am Heart J 2019; 216: 30-41.

3. Cannon CP, Khan I, Klimchak AC, Reynolds MR, Sanchez RJ, Sasiela WJ. Simulation of Lipid-Lowering Therapy Intensification in a Population with Atherosclerotic Cardiovascular Disease. JAMA Cardiol 2017; 2(9): 959-966.

4. Rahimi K, Bidel Z, Nazarzadeh M, Copland E, Canoy D, Ramakrishnan R, et al., Pharmacological blood pressure lowering for primary and secondary prevention of cardiovascular disease across different levels of blood pressure: an individual participant-level data meta-analysis. The Lancet 2021; 397: 1625-1636.

5. Python Software Foundation. Python Language Reference, version 3.9. Available at <http://www.python.org>.

6. Law MR, Wald NJ, Morris JK, Jordan RE. Value of low dose combination treatment with blood pressure lowering drugs: analysis of 354 randomised trials. Br Med J 2003; 326: 1427.
